# Mining drug–target interactions from biomedical literature using chemical and gene descriptions-based ensemble transformer model

**DOI:** 10.1093/bioadv/vbae106

**Published:** 2024-07-22

**Authors:** Jehad Aldahdooh, Ziaurrehman Tanoli, Jing Tang

**Affiliations:** Research Program in Systems Oncology, Faculty of Medicine, University of Helsinki, Helsinki 00290, Finland; Doctoral Programme in Computer Science, University of Helsinki, Helsinki 00290, Finland; Institute for Molecular Medicine Finland, University of Helsinki, Helsinki 00290, Finland; BioICAWtech, Organization, Helsinki 00290, Finland; Research Program in Systems Oncology, Faculty of Medicine, University of Helsinki, Helsinki 00290, Finland

## Abstract

**Motivation:**

Drug–target interactions (DTIs) play a pivotal role in drug discovery, as it aims to identify potential drug targets and elucidate their mechanism of action. In recent years, the application of natural language processing (NLP), particularly when combined with pre-trained language models, has gained considerable momentum in the biomedical domain, with the potential to mine vast amounts of texts to facilitate the efficient extraction of DTIs from the literature.

**Results:**

In this article, we approach the task of DTIs as an entity-relationship extraction problem, utilizing different pre-trained transformer language models, such as BERT, to extract DTIs. Our results indicate that an ensemble approach, by combining gene descriptions from the Entrez Gene database with chemical descriptions from the Comparative Toxicogenomics Database (CTD), is critical for achieving optimal performance. The proposed model achieves an *F*1 score of 80.6 on the hidden DrugProt test set, which is the top-ranked performance among all the submitted models in the official evaluation. Furthermore, we conduct a comparative analysis to evaluate the effectiveness of various gene textual descriptions sourced from Entrez Gene and UniProt databases to gain insights into their impact on the performance. Our findings highlight the potential of NLP-based text mining using gene and chemical descriptions to improve drug–target extraction tasks.

**Availability and implementation:**

Datasets utilized in this study are accessible at https://dtis.drugtargetcommons.org/.

## 1 Introduction

The study of small molecules and their interactions with protein targets is a key element in drug discovery research. Over the years, a vast amount of information about these interactions has been published in scientific literature including high throughput screening studies (Anastassiadis *et al.* 2011, Davis *et al.* 2011). With recent advancements in natural language processing (NLP) technologies, there is an ever-increasing need to develop semi-automated text-based transformer models (such as BERT) that can dig further into the text of a screening study to mine DTIs subject to human approval (Aldahdooh *et al.* 2022). In the field of NLP, entity-relationship extraction is a process of identifying and extracting meaningful relationships between entities (in this case compounds and protein targets) from textual data. To accelerate the extraction of drug–target interactions (DTIs), the BioCreAtIvE (Critical Assessment of Information Extraction systems in Biology) consortium organized a DrugProt challenge (Miranda *et al.* 2021), which provided participants with a manually annotated dataset, the DrugProt corpus, which contains PubMed abstracts, manually annotated chemical compound and gene/protein mentions, and their interactions of 13 types that cover key relations of biomedical importance. Most of the participants in the DrugProt challenge applied the most recent pre-trained models like BERT to train on the domain of DTIs. For example, in Weber *et al.* (2022), authors leveraged ensembles of pre-trained language models such as RoBERTa-large (Liu *et al.* 2019), using chemical descriptions to achieve an *F*1 score of 79.73 as the best model in the DrugProt challenge. More recently, authors in Luo et al. (2022a) employed a sequence labeling framework, achieving a state-of-the-art *F*1 score of 80.0 on the test set. Other related works also include a BioBERT-based method (McInnes *et al.* 2024) and a transformer-based method (Sarrouti *et al.* 2022). In a recent study (Iinuma *et al.* 2022), the authors proposed to enhance the supervised extraction of DTIs by integrating distantly supervised models. While traditional supervised methods rely on manually labeled data for training, which can be resource-intensive and time-consuming, distantly supervised models utilize existing knowledge bases to label the data automatically. However, their experimental results revealed that the proposed approach could have improved the performance accuracy as expected. On the contrary, the inclusion of mislabeled data resulted in even lower performance accuracy. Therefore, this study highlights the limitations within the current supervised approach to DTIs extraction.

In light of the challenges faced by the supervised extraction approaches, an alternative strategy in Asada *et al.* (2023) has emerged which emphasizes the integration of heterogeneous knowledge graphs (HKGs) into drug–drug interaction extraction from biomedical literature. Recognizing the necessity of incorporating a diversity of background knowledge into the extraction process, like human experts comprehend and dissect the scientific literature, the authors built the drug representations by conducting a link prediction task on a pharmaceutical HKG dataset. Then, they combined the contextual information of the input sentences in the corpus with the drug information from the HKG dataset. Their method achieved an *F*1-score of 77.9 on the development set, which is a DrugProt subset provided by the competition organizer to evaluate the model’s performance during the development phase.

In this study, we delve into a distinctive aspect of gene descriptions sourced from the Entrez Gene database—an avenue not previously explored, as most research primarily focuses on utilizing information from protein databases such as UniProt (Coudert *et al.* 2023). Additionally, we pioneered the combined use of both chemical and gene descriptions. To achieve this, we deployed a Multi-Model Ensemble (CGDE) that combines specialized models focused on analysing chemical and gene data. Our work not only improves the *F*1 scores achieved by the aforementioned related works but also highlights the importance of incorporating gene descriptions in the DTI extraction process. By leveraging the rich information in gene descriptions, our method demonstrates a more comprehensive understanding of the intricate relationships between drugs and their target proteins, ultimately contributing to more efficient and accurate DTI extraction from biomedical text.

## 2 Methods

### 2.1 Overview of the workflow

**Figure 1.**
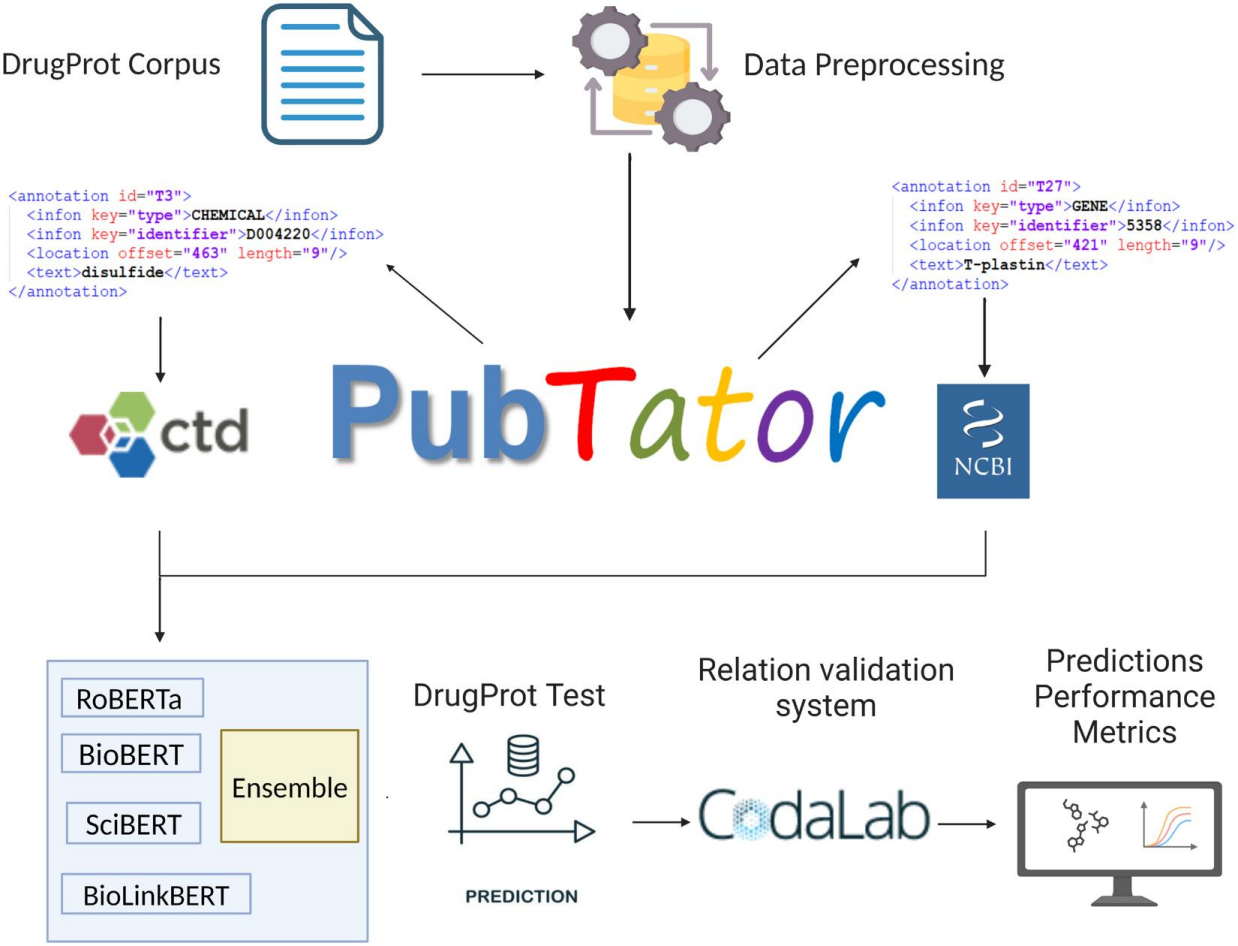
Workflow for mining drug–target interactions using the ensemble transformer model. The process begins with the DrugProt corpus, which is preprocessed to extract relevant annotations for chemicals and genes. These annotations are then integrated using PubTator, a tool that facilitates the identification of biomedical entities including genes and chemicals. The annotations are further categorized and linked to the Comparative Toxicogenomics (CTD) and NCBI databases. The preprocessed data is then fed into an ensemble of pre-trained transformer models, including RoBERTa, BioBERT, SciBERT, and BioLinkBERT, to predict drug–target interactions. The ensemble model’s predictions are validated using the DrugProt hidden test set to assess the final model performance, with results submitted to the CodaLab platform for official evaluation. This workflow aims to enhance the extraction and prediction accuracy of drug–target interactions from biomedical literature.

To provide a clear and intuitive understanding of our methodology, we have included a flowchart (Fig. 1) that outlines the key steps and components of our study. This visual representation aims to enhance comprehension of the processes involved in mining DTIs using an ensemble transformer model.

### 2.2 Datasets

**Figure 2.**
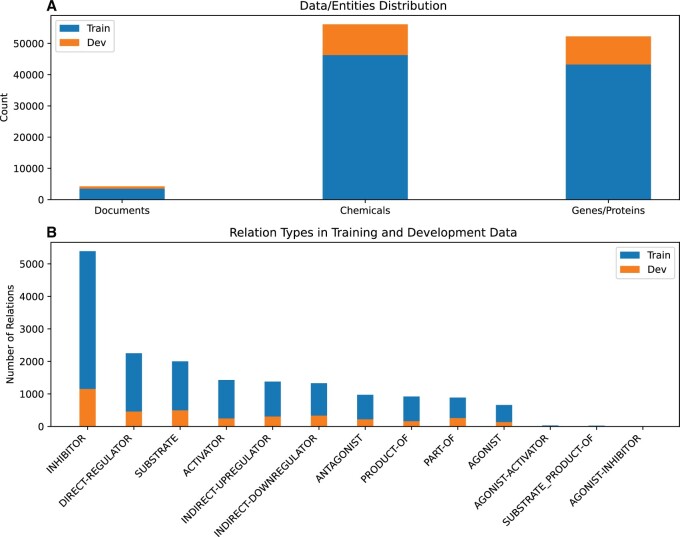
Distribution of the biological entities in the DrugProt dataset. (A) Number of documents (PubMed articles), chemical and gene entities. (B) Number of relation types in DTIs.

We have mainly used DrugProt for training the models. DrugProt is a publicly available dataset containing manually annotated entities and relations relevant to drug discovery research. The DrugProt dataset is divided into training, development, and testing subsets. Training and development subsets were provided to the participants for developing their models, while the testing data remained hidden to ensure unbiased evaluation. After the development stage, participants can submit their predictions on the testing data to the Codelab platform and receive the final prediction performance. Figure 2 provides a comprehensive overview of the statistics for the DrugProt training and development datasets.

Within this dataset, gene entities are categorized into two primary classifications: GENE-Y, which represents normalizable genes (i.e. genes that can be linked to a specific biological database), and GENE-N, marking non-normalizable genes. In terms of relation types, the DrugProt dataset is quite extensive, featuring 13 distinct categories, including: *INDIRECT-DOWNREGULATOR*, *INDIRECT-UPREGULATOR*, *DIRECT-REGULATOR*, *ACTIVATOR*, *INHIBITOR*, *AGONIST*, *ANTAGONIST*, *AGONIST-ACTIVATOR*, *AGONIST-INHIBITOR*, *PRODUCT-OF*, *SUBSTRATE*, *SUBSTRATE_PRODUCT-OF*, and *PART-OF*. These classifications enable a comprehensive understanding of the relations and interactions among various entities within the DrugProt dataset.

The most prevalent relation type is INHIBITOR, with 5388 instances, followed by DIRECT-REGULATOR with 2247 instances in the training dataset, as shown in Fig. 2. The least represented relation types are AGONIST-INHIBITOR with 13 instances, SUBSTRATE_PRODUCT-OF with 24 instances, and AGONIST-ACTIVATOR with 29 instances in the training dataset.

We conducted experiments using various model configurations, and subsequently linked chemicals and genes to their ontologies. To ensure consistency, we adopted the same unique gene identifiers as in Weber *et al.* (2022) for both the training and development datasets. These identifiers were normalized using a BioSync model (Sung *et al.* 2020) for chemicals, leveraging the comprehensive BioCreative V CDR (BC5CDR) dataset (Li *et al.* 2016), and for proteins, utilizing the BioCreative II Gene Normalization (BC2GN) dataset (Morgan *et al.* 2008).

To retrieve chemical and gene entities for the hidden test dataset, we utilized Pubtator’s API (Wei *et al.* 2019), which utilizes GNormPlus (Wei *et al.* 2015) for the gene annotation and determines their NCBI Gene identifiers. For the chemical annotation, Pubtator employs TaggerOne (Leaman and Lu 2016) to link the chemical names to MeSH identifiers. We have also employed the BioSync method (Sung *et al.* 2020) to perform the normalization, using the BC2GN-Gene (dmis-lab/biosyn-sapbert-bc2gn) model, which was applied to the BC2GN dataset. Using the gene IDs, we obtained the gene summary descriptions from the Entrez Gene database, making our experiment the first attempt to acquire such information.

For example, the Entrez Gene entry for ANGPT2 (Angiopoietin 2) provides a comprehensive summary:

This gene belongs to the angiopoietin family of growth factors. The protein encoded by this gene is an antagonist of angiopoietin 1, and both angiopoietin 1 and angiopoietin 2 are ligands for the endothelial TEK receptor tyrosine kinase. Angiopoietin 2 is upregulated in multiple inflammatory diseases and is implicated in the direct control of inflammation-related signaling pathways. The encoded protein affects angiogenesis during embryogenesis and tumorigenesis, disrupts the vascular remodeling ability of angiopoietin 1, and may induce endothelial cell apoptosis. This gene serves a prognostic biomarker for acute respiratory distress syndrome. [provided by RefSeq, Aug 2020]

This summary includes gene function, role in disease, and interactions with other proteins, providing valuable context for understanding drug–gene interactions. Furthermore, we extracted the description for each gene from their respective web pages using specific HTML tags.

For the protein annotation, we obtained the descriptions from the UniProt database. For example, the UniProt entry for ANGPT2 (O15123) provides detailed protein-specific information:

ANGPT2_HUMAN (O15123) – Binds to TEK/TIE2, competing for the ANGPT1 binding site, and modulating ANGPT1 signaling. Can induce tyrosine phosphorylation of TEK/TIE2 in the absence of ANGPT1. In the absence of angiogenic inducers, such as VEGF, ANGPT2-mediated loosening of cell-matrix contacts may induce endothelial cell apoptosis with consequent vascular regression. In concert with VEGF, it may facilitate endothelial cell migration and proliferation, thus serving as a permissive angiogenic signal. Involved in the regulation of lymphangiogenesis.

While detailed, the UniProt description focuses narrowly on the protein’s specific functions and modifications. Both the NCBI and UniProt databases provide information about the role of genes in different diseases or pathways and their interactions with other proteins. However, NCBI additionally provides gene family information, and the amount of textual descriptions returned by NCBI is slightly greater than UniProt. This could be one of the reasons for the superiority of gene descriptions in NCBI over those in the UniProt database.

In our study, we utilized the same chemical descriptions as in Weber *et al.* (2022), which were derived from the Comparative Toxicogenomics Database (CTD). These descriptions were obtained from the definition field of the CTD’s chemical vocabulary, specifically taking the first sentence of the definition. Furthermore, we computed 576 descriptors from the protein sequences using amino acid index database (Kawashima and Kanehisa 2000). From these, 420 are based on monopeptide and dipeptide frequencies for the 20 amino acids. The remaining 156 features relate to various physicochemical properties, mass, length, and free energy. Length corresponds to the total number of amino acids present in the FASTA sequence of a protein target. To facilitate a comprehensive understanding and replication of our computational methodology, we have developed a Python script that automates the extraction and calculation of these descriptors. The script employs the Biopython library (Cock *et al.* 2009) to analyse protein sequences, calculating a broad array of features such as molecular weight, aromaticity, hydrophobicity, flexibility, polarizability, free energy, and steric properties, among others. It includes functions for counting amino acids, computing their percentages in sequences, and assessing protein stability through the instability index, gravy (grand average of hydropathicity), and isoelectric point calculations. This script is accessible *via* our GitHub repository (https://github.com/JehadAldahdooh/BioSeqFeatureExtraction). By detailing the computational steps and parameters used to derive our dataset’s descriptors, we aim to enhance the reproducibility and transparency of our research methodology.

### 2.3 Methods

We approached the drug–target relation classification task as a multi-label problem similar to Weber *et al.* (2022). However, we employed various setup configurations, including the use of focal loss instead of binary cross-entropy loss. Furthermore, we incorporated both gene and chemical description datasets. We utilized various pre-trained BERT (Bidirectional Encoder Representations from Transformers) models (large and base), including SciBERT (Beltagy *et al.* 2019), BioBERT (Lee *et al.* 2020), RoBERTa (Liu *et al.* 2019), and BioLinkBERT (Yasunaga *et al.* 2022), with a primary emphasis on the RoBERTa model. We combined all these models to form an ensemble of different transformer models. For example, in the sentence-based ensemble (SBE) model, we used RoBERTa as the main BERT variant, while in other models, we used different BERT variants, including:

- **SciBERT** (Beltagy *et al.* 2019) is a scientific text-specific version of BERT, fine-tuned on an expansive corpus of scientific papers from Semantic Scholar, encompassing diverse scientific disciplines. SciBERT is designed to better capture and understand the language and terminology used in scientific literature. In our experiments, we employed the allenai/scibert_scivocab_uncased model, which is publicly available through the Hugging Face Hub (https://huggingface.co/allenai/scibert_scivocab_uncased).
- **BioBERT** (Lee *et al.* 2020) offers a specialized language representation model for the biomedical domain, trained on large-scale biomedical corpora, available at dmis-lab/biobert-large-cased-v1.1 (https://huggingface.co/dmis-lab/biobert-large-cased-v1.1).
- **RoBERTa** (robustly optimized BERT) is a variant of the BERT model developed by Facebook AI and is designed to improve upon the original BERT model in several ways by using a larger and more diverse training dataset, longer training time, and dynamic masking to improve the model’s ability to understand natural languages. RoBERTa also removes the next sentence prediction objective used in BERT, which was found to be less important for many NLP tasks. RoBERTa-large is a variant of the RoBERTa model, with even more parameters and a longer training time. It is designed to further improve upon the original RoBERTa model’s ability to understand natural languages. RoBERTa-large is generally considered to be more powerful and accurate than the smaller RoBERTa model. We used the RoBERTa-large-PM-M3-Voc (Lewis *et al.* 2020) (https://github.com/facebookresearch/bio-lm) model in our experiments.
- **BioLinkBERT** (Yasunaga *et al.* 2022) is a unique version of BERT that includes document link knowledge, such as hyperlinks and citation links from PubMed abstracts, allowing the model to integrate information from multiple documents. Pre-training involves feeding linked documents into the same language model context, in addition to a single document. In our experiments, we employed the BioLinkBERT-large model that is available at https://huggingface.co/michiyasunaga/BioLinkBERT-large.

**Figure 3.**
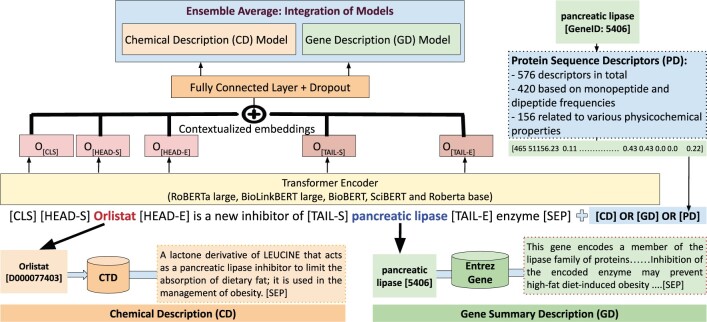
Overview of the best-performing ensemble model. Our experiments utilize an ensemble of the RoBERTa-large-PM-M3-Voc, BioBERT-v1.1, BioLinkBERT-large, SciBERT, and Roberta Base models. The abstract texts are processed with BERT, together with target gene and chemical descriptions extracted from the Entrez and CTD databases, respectively. Furthermore, we integrated 576 protein sequence-based features. Special tokens mark the start and end of chemical-gene entities. These tokens are also incorporated in the model’s input.

To generate the training and development instances from an abstract, we considered all compound-target pairs that occur in the same sentences. We created one instance for each compound-target entity pair, inserting special tokens [HEAD-S], [HEAD-E], [TAIL-S], and [TAIL-E] to mark the start and end of the head (the chemical entity) and tail (the target gene entity). We used the context embeddings of the [CLS] token from the last hidden layer of the BERT model and fed it to a fully connected layer to obtain the logits. Additionally, we experimented with the embeddings of the target gene features, concatenating them with the [CLS] output. The main framework is shown in Fig. 3. Moreover, we incorporated an experimental strategy of varying random seeds to assess their impact on the model’s stability and performance (Sellam *et al.* 2021, Weber *et al.* 2022). In some cases of random seeds, the model failed to converge, highlighting the sensitivity of our approach to initial conditions. This underlines the importance of careful selection of random seeds in reproducibility studies.

### 2.4 Loss function

We used focal loss, which is a loss function designed to address the issue of class imbalance in classification tasks. Focal loss was first introduced in Lin *et al.* (2017) to improve the performance of object detection models. However, it can also be applied to our classification problem where there is a class imbalance between the relations. The focal loss is an extension of the binary cross-entropy (BCE) loss, but it introduces a modulating factor that decreases the contribution of well-classified examples to the loss. This helps the model to focus more on the hard-to-classify examples, thus addressing the class imbalance issue. Focal loss has two main parameters:

- **Gamma (*γ*):** The focusing parameter, which determines how much the contribution of well-classified examples is reduced. A higher value of gamma will lead to a more focused loss function. In our study, we set the focusing parameter to γ=0.5.
- **Eps (*ϵ*):** A small value added to the logarithm term to prevent division by zero or taking the logarithm of zero. We used the default value 1e−6.

The focal loss is defined as:

(1) $$\begin{array}{c} \text{loss}=-\sum_{i=1}^{N}[y_{i}\cdot {\left(1-p_{i}\right)}^{\gamma }\cdot \;\text{log}(p_{i}+\epsilon )\\ +(1-y_{i})\cdot p_{i}^{\gamma }\cdot \;\text{log}(1-p_{i}+\epsilon )] \end{array}$$

where

 *N* is the total number of samples in the dataset,

 *y*_i_ is the true label for the *i*th sample,

 *p*_i_ is the predicted probability for the *i*th sample.

### 2.5 Evaluation metrics

To evaluate the performance of our models, we utilized the micro-averaged *F*1 score. The *F*1 score, defined as the harmonic mean of precision and recall, provides a single metric that balances both false positives (FP) and false negatives (FN). The formula for the *F*1 score is:

(2) $$F1=2\times \frac{\text{Precision}\times \text{Recall}}{\text{Precision}+\text{Recall}}$$

The micro-averaged *F*1 score aggregates the contributions of all classes to compute a global metric. Specifically, it calculates the global precision and recall by summing the true positives (TP), FP, and FN across all classes. The aggregated values are then used to compute the micro-averaged *F*1 score.

The formulas for micro-averaged precision, recall, and *F*1 score are as follows:

(3) $$\text{Micro precision}=\frac{\sum \text{TP}}{\sum \left(\text{TP}+\text{FP}\right)}$$

(4) $$\text{Micro recall}=\frac{\sum \text{TP}}{\sum \left(\text{TP}+\text{FN}\right)}$$

(5) $$\text{Micro}\;F1=2\times \frac{\text{Micro precision}\times \text{Micro recall}}{\text{Micro precision}+\text{Micro recall}}$$

In our study, we used the official DrugProt evaluation library (https://github.com/tonifuc3m/drugprot-evaluation-library) to compute the micro-averaged *F*1 score on the hidden test set.

### 2.6 Experiments

We ran the experiments with 10 different random seeds and averaged the results to obtain a more robust estimate of the prediction performance. To assess the effectiveness of our approach, we conducted a series of four experiments. Subsequently, we submitted the final predictions to the official site for the evaluation on the hidden test set. The experiments conducted are summarized as follows:

- **Model 1 (Base model): SBE model**
  In this experiment, we trained an ensemble model consisting of 10 RoBERTa-large-PM-M3-Voc models, with different seeds. We exclusively utilized sentences without any external chemical or gene descriptions.
- **Model 2: gene description ensemble (GDE) model**
  The main goal of this experiment was to assess how gene descriptions affect prediction accuracy. To achieve this, we constructed a GDE comprising 10 models. These models encompassed different models to leverage their unique strengths—consisting of the Roberta Base, SciBERT, RoBERTa-large-PM-M3-Voc, BioLinkBERT-large, and BioBERT-v1.1 single models—all of which incorporated gene descriptions. By including a diverse range of model architectures and learning strategies within the GDE, we aimed to provide extensive coverage and enhance the ensemble’s overall performance. The ensemble model is constructed by taking the average of the probabilities generated by the models with a threshold of 0.5 or higher.
- **Model 3: chemical description ensemble (CDE) model**
  To establish the benchmark for our comparative analysis, we replicated the top-performing model from the DrugProt challenge, which achieved an *F*1 score of 79.7 on the concealed DrugProt test data. This result was attained by training an ensemble of ten RoBERTa-large-PM-M3-Voc models, incorporating chemical definitions from the CTD for both the training and development sets. We employed the same approach by developing an ensemble of 10 RoBERTa-large-PM-M3-Voc models with different seeds. The ensemble model we employed—consisting of the RoBERTa-large-PM-M3-Voc and BioLinkBERT-large single models.
- **Model 4: chemical and GDE (CGDE) model**
  We employed an ensemble that utilized both gene and chemical description models as an integration to models 1 and 2.
- **Model 5: sequence-based features and text description ensemble (SFTDE) model**
  Protein sequence-based features were incorporated in this experiment. An ensemble of 10 RoBERTa-large-PM-M3-Voc models with different seeds has been constructed using gene descriptions, along with protein sequence-based features. Nevertheless, they did not contribute to an improvement in the results. In order to preprocess the protein sequence-based features, L2 normalization was applied by dividing each vector by its L2 norm, as Normalized Vector = Vector/‖Vector_2_‖, where normalized vector represents the resulting vector after applying the normalization process, and ‖Vector_2_‖ represents the L2 norm of the original vector.

## 3 Results

### 3.1 Performance of the five different models on the hidden DrugProt test data

**Figure 4.**
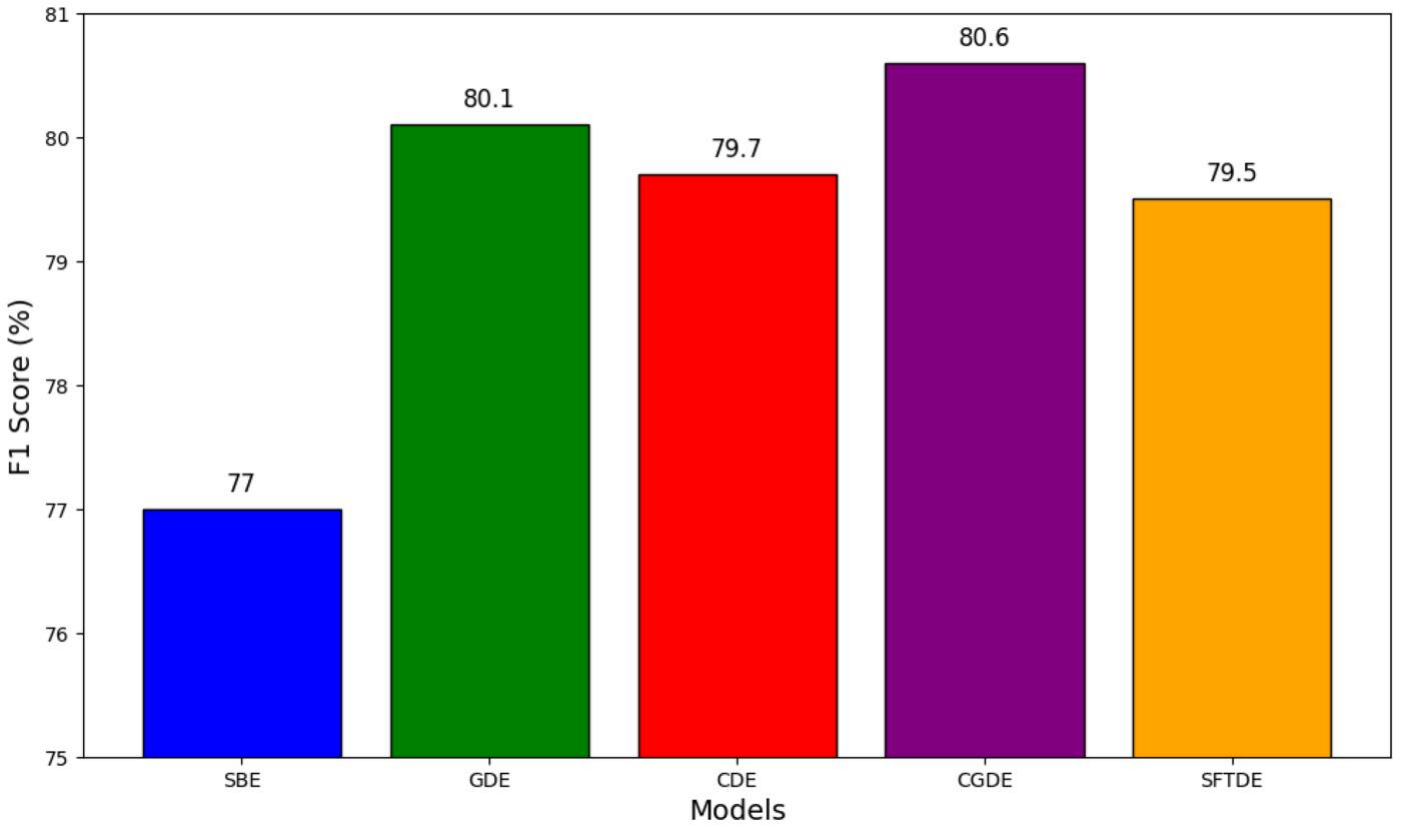
Performance comparison of the five models on the hidden DrugProt test set, presented as micro-averaged *F*1 scores.

The five models demonstrated varying levels of performance on the DrugProt hidden test data. The CGDE model achieved the highest *F*1 score of 80.6, followed by the GDE model with 80.1, the CDE model with 79.7, and the SFTDE model with 79.5. Additionally, the SBE model exhibited an *F*1 score of 77. These results indicate that the CGDE model outperformed the other models in terms of overall predictive accuracy (Fig. 4).

Our experiments showed that utilizing only the Entrez Gene descriptions (GDE model) yielded better performance, with an improvement by approximately 0.4 *F*1 compared to the CDE baseline model. Combining chemical and gene descriptions led to a more significant increase, with approximately 0.9 points *F*1 improvement over the CDE baseline model. These results suggested that the integration of chemical and gene descriptions in an ensemble BERT model achieved the best performance, compared to the top-performing model from the DrugProt challenge. However, adding protein sequence-based features besides gene descriptions led to a slight decrease of approximately 0.2 points *F*1, suggesting that the numeric features may not be optimal when combined with BERT contextual embeddings. Furthermore, our preliminary experiments suggest that an ensemble model derived from identical single models with different random seeds and trained with binary cross-entropy yielded better performance on the DrugProt test set than combining different types of single models. However, this is not the case when using focal loss. This indicates that the choice of loss function plays a crucial role in optimizing model performance (Liu *et al.* 2021).

**Figure 5.**
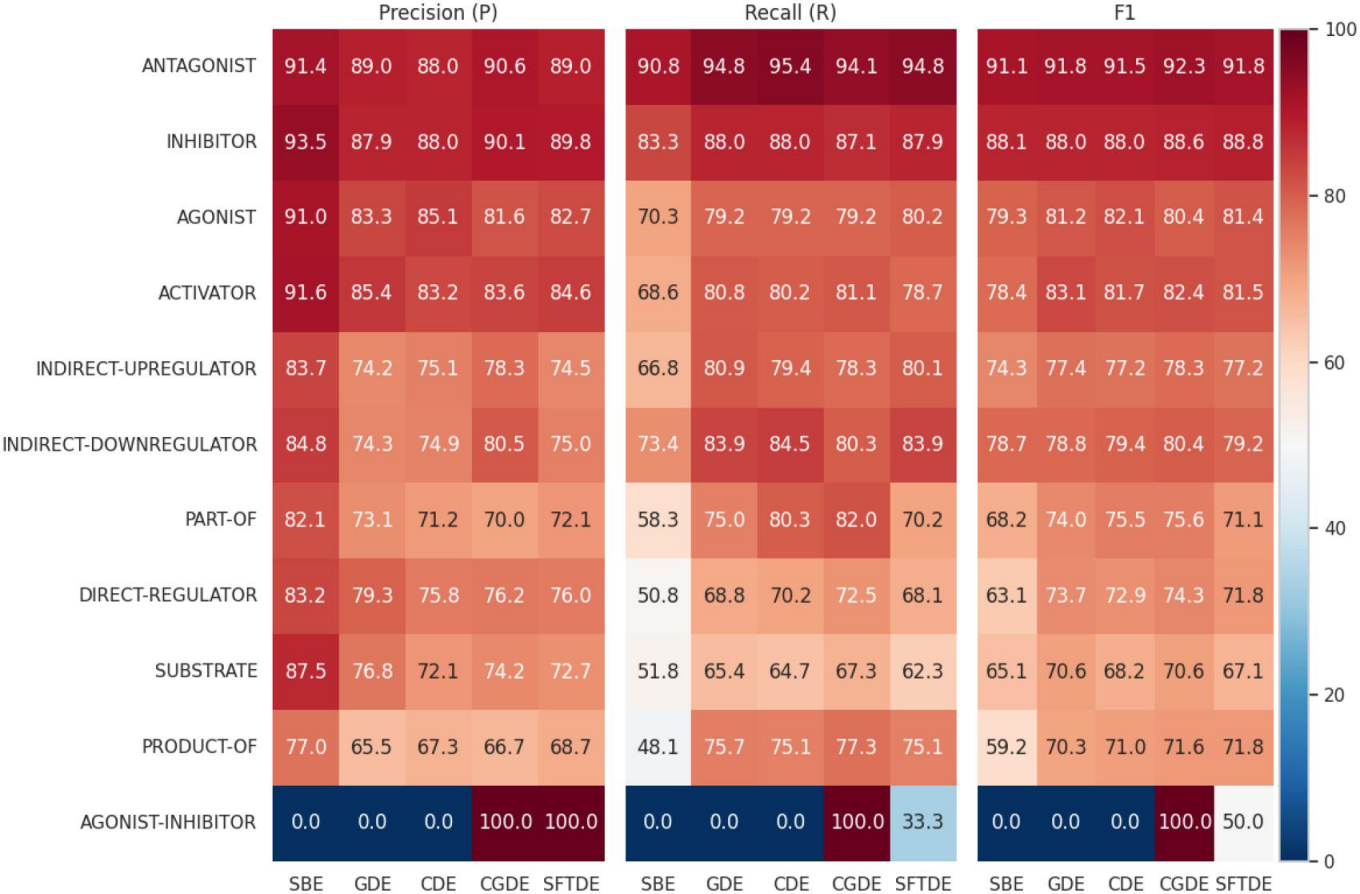
Comparative analysis of model performance per relation types.

In Fig. 5, we compared the performance per class achieved by the five different models. It can be shown that the ANTAGONIST relation seems to consistently have the highest *F*1 score across all models, indicating that this class was the easiest for the models to predict correctly. The highest *F*1 score is 92.3 by the CGDE model. Furthermore, both the ANTAGONIST and INHIBITOR interaction types display a stable and consistent performance across all the five models. This could suggest that the features for these types of interactions are robust and well-captured, which consistently rank in the top two in terms of precision, recall, and *F*1 scores across all the models. The CGDE model generally performs the best across different interaction types. It achieves the highest *F*1 score for ANTAGONIST, DIRECT-REGULATOR, SUBSTRATE, PART-OF, INDIRECT-UPREGULATOR, INDIRECT-DOWNREGULATOR, and AGONIST-INHIBITOR. However, the SFTDE model performs better for INHIBITOR and PRODUCT-OF relations.

### 3.2 Performance on rare classes

The AGONIST-INHIBITOR relation shows the most variability in accuracy across different models since it is rarely present (Fig. 2). For example, the SFTDE model has an *F*1 score of 50, CGDE has an *F*1 score of 100, while SBE, GDE, and CDE have an *F*1 score of 0. This discrepancy could be due to the different ways these models handle this specific interaction class.

In the case of CGDE, we implemented a lower threshold (≥0.2 instead of ≥0.5) to detect this relation. This adjustment was necessary due to its relatively low representation in the training and development dataset, as well as the model’s tendency to predict this relation with a lower probability. Conversely, in the SFTDE model, the gene features appear to aid in capturing the context of the agonist-inhibitor relation, allowing the model to identify it without altering the threshold. Additionally, none of the five models predicts AGONIST-ACTIVATOR or SUBSTRATE_PRODUCT-OF relations, primarily due to the limited size of training and development samples available for these specific relationships.

### 3.3 Recall and precision trade-off

There is an evident trade-off between precision and recall in the five different models. For example, in the DIRECT-REGULATOR relation, the SBE model has a high precision of 83.2 but a relatively low recall of 50.8. In contrast, the CGDE model has a lower precision of 76.2 but a higher recall of 72.5. This indicates that SBE is more conservative in predicting DIRECT-REGULATOR, resulting in fewer FP but more FN. The CGDE, on the other hand, makes more positive predictions, leading to fewer FN but more FP.

### 3.4 Performance comparison with the latest top-performing model on DrugProt

According to the DrugProt article, 30 teams from four continents were participating in the competition (Miranda-Escalada *et al.* 2023). The top three models in the DrugProt challenge were Humboldt (CDE) (Weber *et al.* 2022), KU-AZ (Yoon *et al.* 2021), and NCBI (Luo *et al.* 2021) teams. After the DrugProt challenge, more models have been submitted, with one of them outperforming the original top-performing CDE model. The latest top-performing model was achieved after the competition by the NLM-NCBI team, who utilized an ensemble approach employing sequence labeling models with standard loss that were trained using DevES and TrainES *via* majority voting (Luo *et al.* 2022a). Based on the results of the hidden test data, our CGDE model has achieved higher overall accuracy compared to the NLM-NCBI model (Table 1). The CGDE model presents several unique ensemble approaches that distinguish our work from other models evaluated in the DrugProt test set:

**Table 1.** Performance comparison between the KU-AZ, NLM-NCBI, Humboldt, NLM-NCBI Ensemble model, and our best model (CGDE) on the DrugProt test data.

Relation type	KU-AZ	NLM-NCBI	Humboldt	NLM-NCBI-ensemble	Our best model (CGDE)
ANTAGONIST	–	92.7	91.5	92.4	92.3
INHIBITOR	87.8	86.7	88.0	87.6	88.6
AGONIST	–	84.3	82.1	85.3	80.4
ACTIVATOR	–	83.0	81.7	83.2	82.4
INDIRECT-UPREGULATOR	–	77.5	77.2	78.1	78.3
INDIRECT-DOWNREGULATOR	–	75.7	79.4	75.9	80.4
PART-OF	–	77.0	75.5	76.9	75.6
DIRECT-REGULATOR	66.7	71.1	72.9	70.6	74.3
SUBSTRATE	68.3	72.2	68.2	73.4	70.6
PRODUCT-OF	–	68.3	71.0	68.2	71.6
AGONIST-INHIBITOR	–	100.0	0.0	100.0	100.0
Overall-*F*1	78.9	79.5	79.7	80.0	80.6

- **NCBI gene summary data:** Unlike other studies that used UniProt gene function text summaries, our model utilizes gene text descriptions from the NCBI Entrez Gene database. This choice was based on our findings that the NCBI gene summaries provided more comprehensive information, leading to better model performance.
- **Use of gene and chemical text descriptions:** Our ensemble model uniquely incorporates gene descriptions from the Entrez Gene database and chemical descriptions from the CTD. This integration allows our model to leverage additional contextual information, improving its ability to accurately predict drug-protein interactions.

In assessing our CGDE ensemble model, alongside the KU-AZ, NLM-NCBI, Humboldt (CDE) ensemble model, and the NLM-NCBI ensemble model, we observed a significant variance in the class-wise results. For the ANTAGONIST class, our model demonstrated high efficacy, with a success rate of 92.3. This was nearly on par with the NLM-NCBI models, but markedly superior to that of the Humboldt CDE model, which scored 91.5. In the INHIBITOR class, our CGDE model outstripped the competition, achieving a score of 88.6. This surpassed the NLM-NCBI ensemble model’s 87.6, KU-AZ with 87.8, and the 88 score of the Humboldt CDE model. This pattern was mirrored in the INDIRECT-DOWNREGULATOR, DIRECT-REGULATOR, and PRODUCT-OF relations categories.

However, a different trend emerged for the AGONIST class. Our model lagged slightly behind at 80.4, compared to the NLM-NCBI and Humboldt ensemble models. Similarly, in the ACTIVATOR class, while our model outpaced the Humboldt ensemble model, it was slightly behind the NLM-NCBI ensemble model at 82.4. In the INDIRECT-UPREGULATOR relation class, our model performed comparably with the NLM-NCBI model, with a marginal 0.2 *F*1 points advantage, and surpassed the Humboldt ensemble model. Lastly, in the AGONIST-INHIBITOR class, both models achieved a flawless score of 100. Notably, we found that our model achieved better performance on the UP/DOWN-REGULATOR categories, suggesting that the gene descriptions may contain important information for predicting these relation types more accurately.

Despite the class-specific variations, the overall *F*1 score for our CGDE ensemble model narrowly topped the list with an overall score of 80.6, compared to the NLM-NCBI model’s 80.0 and Humboldt CDE model’s 79.7.

### 3.5 Comparison of NCBI Entrez Gene summary and UniProt protein function description—subsection head

The UniProt protein function is a feature available in the UniProt database (Coudert *et al.* 2023), a comprehensive resource for protein sequence and functional information. The protein function section provides a brief and informative summary of the protein’s function including the protein’s biological activity, localization, interaction partners, and related pathways. The Humboldt team utilized multiple single models using RoBERTa-large-PM-M3-Voc with different seeds, in conjunction with an ensemble model that integrated UniProt protein function descriptions. Their testing ground was the DrugProt development dataset and reported their single results as the mean and standard deviation of the top three runs from a pool of five different random seeds. For our part, we employed a similar methodology, using a variety of single models using RoBERTa-large-PM-M3-Voc and an ensemble model. However, our findings indicated that the NCBI Entrez Gene summary data offered a more comprehensive resource, and consequently resulted in slightly better *F*1 scores when contrasted with the UniProt protein function (Table 2). The results demonstrate that a single model employing gene summary description attains a superior *F*1 score, showing an improvement of +0.7 points, in contrast to the performance of three model runs that utilized UniProt protein function.

**Table 2.** Comparative analysis of including the UniProt protein function summaries and the NCBI Entrez Gene summary data.

	Single			Ensemble		
	*P*	*R*	*F*1	*P*	*R*	*F*1
UniProt protein function (3 runs)	77.4 ± .6	79 ± 0.5	78.5 ± 0.2	78.8	79.6	79.2
NCBI Entrez Gene summary (1 run)	79.8	79	79.4	–	–	–
NCBI Entrez Gene summary (3 runs)	79.9 ± 1.6	77.9 ± 1.7	78.9 ± 0.4	79.6	79.5	79.5

The blank row in the ensemble section indicates that the ensemble model was not evaluated using the NCBI Entrez Gene summary data for the single run. Ensemble results are only shown for three runs where multiple seeds were used. Standard deviation estimates are provided for three runs with different random seeds. For the ensemble model, only mean performance is reported.

### 3.6 Comparative analysis: gene-based descriptor versus sentence-based models

**Figure 6.**
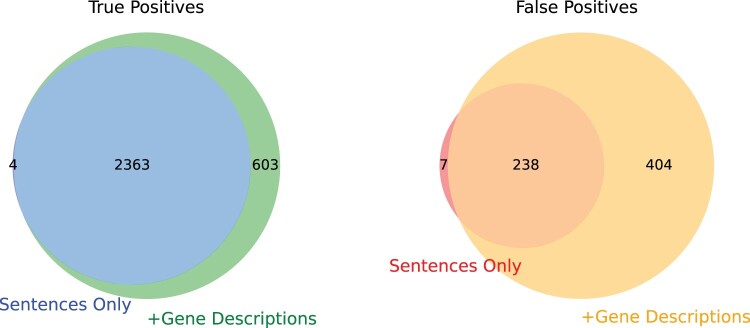
Comparative analysis of true and false positives in sentence-based versus enhanced gene description models.

In our study, we investigated the influence of entity descriptions on predictive performance by comparing two distinct models: the SBE model using sentences only and an extended model incorporating gene descriptions. Our analysis conducted on the development dataset yielded valuable insights into their performance. The results of both ensemble models are provided in Supplementary File 1. As shown in Fig. 6, in terms of TPs, we observed that the extended model with gene descriptions outperformed the sentence-based model, with 2966 TPs compared to 2367 TPs. This suggests that gene descriptions provide a substantial benefit in enhancing the predictive capabilities of the model. However, it is important to note that the extended model also exhibited a higher number of FPs at 642, as opposed to the baseline model’s 245 FPs. This highlights a trade-off between sensitivity and specificity when incorporating gene descriptions.

The overlap in TPs between both models was substantial, with 2363 TPs in common. Notably, the sentence-based ensemble model identified four unique TPs that the extended model did not, while the extended model with gene descriptions identified 603 unique TPs not found by the baseline model. These unique contributions emphasize the added value of including gene descriptions in the model.

We also employed a comprehensive relation type analysis and error distribution analysis. The examination of TPs revealed that the extended model, enriched with gene descriptions, demonstrated substantial improvements across various relation types. Specifically, it surpassed the baseline model in identifying ACTIVATOR and AGONIST relations with 183 TPs and 95 TPs, respectively, compared to the baseline’s 141 and 76. While both models performed comparably in ANTAGONIST relations, the extended model excelled in identifying DIRECT-REGULATOR relations with an impressive 280 TPs compared to the baseline’s 131. Additionally, the extended model outperformed the baseline in detecting INDIRECT-DOWNREGULATOR relations, tallying 265 TPs to the baseline’s 188.

However, this enhancement in TPs came at the cost of increased FPs. The extended model exhibited more FPs in ACTIVATOR, AGONIST, ANTAGONIST, DIRECT-REGULATOR, and INDIRECT-DOWNREGULATOR relations, highlighting a potential trade-off between sensitivity and specificity. Notably, the significant improvement in identifying DIRECT-REGULATOR relations by the extended model underscores the added value of gene descriptions in this specific category. Despite the rise in FPs, our analysis suggests that gene description information proves beneficial, particularly for specific relation types, offering valuable insights into the influence of entity descriptions on our predictive models.

### 3.7 Case study of Ozone and IL-6 interaction

**Figure 7.**
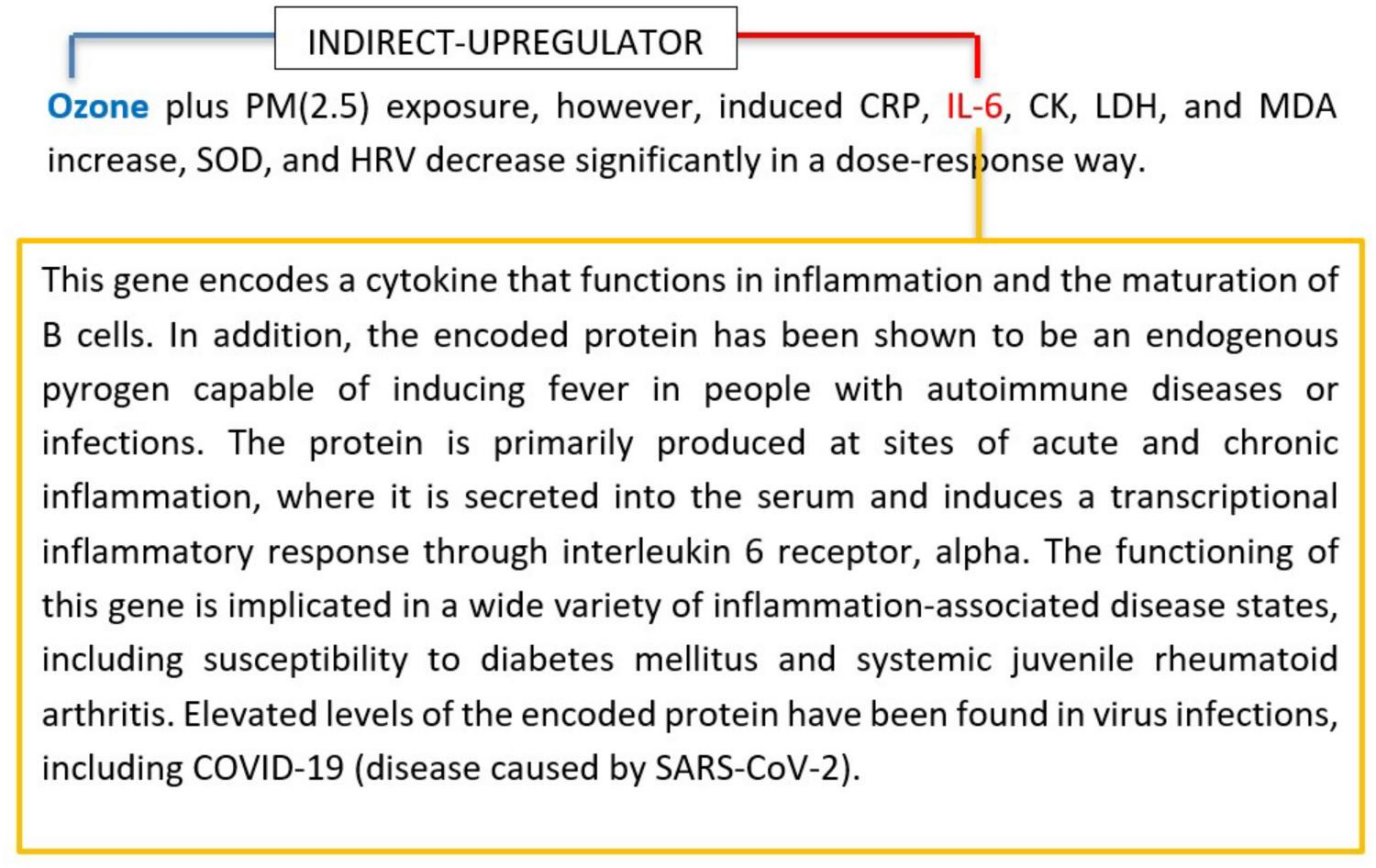
A case study analysis: Ozone and IL-6 interaction. Gene summary description extracted from NCBI Entrez database is highlighted in the rectangle.

In this case study, we delve into the interaction between Ozone and the IL-6 gene, highlighting the differential predictive capabilities of the sentence-based and gene-based descriptor models. The sentence from the dataset that we focus on is shown in Fig. 7. This sentence mentions the effect of Ozone exposure on various genes, including IL-6, but does not explicitly state the nature of the interaction.

We found that the SBE model, relying solely on the sentence, was unable to predict the relationship between Ozone and IL-6 due to the lack of explicit information. In contrast, the GDE model, which incorporates the gene description information, correctly predicted the relationship as INDIRECT-UPREGULATOR. It inferred this interaction by understanding the role of IL-6 in inflammation and its potential upregulation in response to environmental stressors like Ozone.

### 3.8 Case study of attention visualization

**Figure 8.**
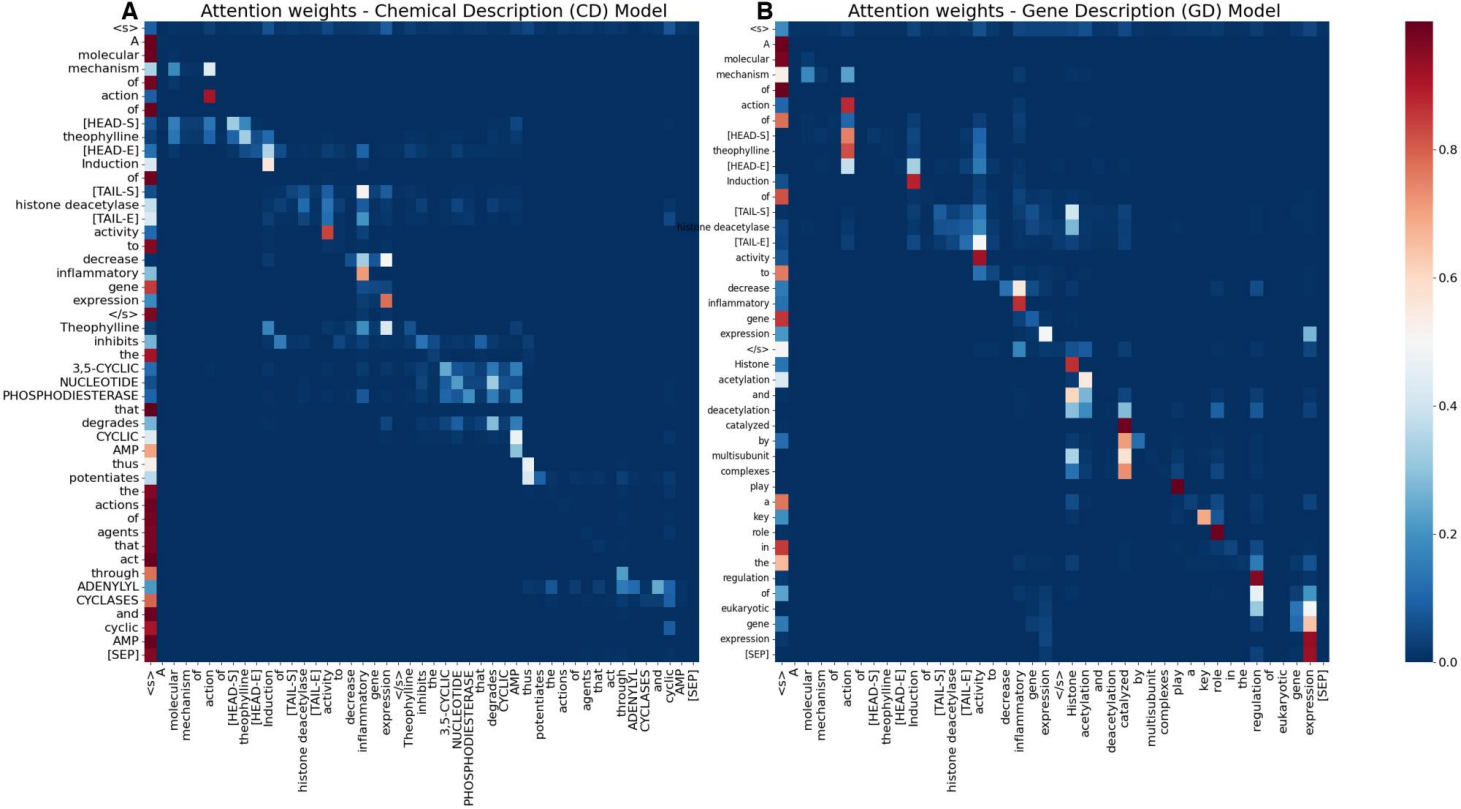
Attention heatmaps of the last layer’s first head for the sentence: “A molecular mechanism of action of theophylline: Induction of histone deacetylase activity to decrease inflammatory gene expression” with (A) chemical description: “Theophylline inhibits the 3,5-CYCLIC NUCLEOTIDE PHOSPHODIESTERASE that degrades CYCLIC AMP thus potentiates the actions of agents that act through ADENYLYL CYCLASES and cyclic AMP”, and (B) gene description: “Histone acetylation and deacetylation, catalysed by multisubunit complexes, play a key role in the regulation of eukaryotic gene expression”. The *x*-axis and *y*-axis represent individual words from the sentence.

To gain an insight into how the GDE and CDE models attend to different parts of the input text, such as gene summary and chemical summary, we visualized the attention distributions of the last layer’s first head for the GDE and CDE models. As shown in Fig. 8, the attention heatmap highlights the regions that the model focuses on when making predictions about DTIs, with brighter shades indicating higher attention weights. Figure 8 illustrates the relationships between tokens in a sentence discussing the molecular mechanisms of gene expression, focusing on histone acetylation (gene entity) and theophylline (chemical entity). The color scale ranges from blue (lower values) to red (higher values), reflecting the degree of interaction or co-occurrence between the tokens.

In the case of the CDE model, significant attention is directed toward tokens such as “inflammatory”, “AMP”, and “Induction” indicating a focus on the biochemical pathways and the role of AMP and the induction process in the action mechanism of theophylline. This focused attention highlights key components relevant to the drug’s mechanism. On the other hand, the GDE model shows higher attention weights on tokens like “catalysed”, “regulation”, “expression”, “histone”, and “activity”. This distribution suggests an emphasis on the genetic and regulatory aspects of histone acetylation and deacetylation, signifying the model’s focus on the processes crucial for gene expression regulation.

The differences in the attention patterns between the GDE and CDE models illustrate how each model uniquely processes and prioritizes the input data to identify crucial features for predicting DTIs.

## 4. Discussion and conclusion

Data deposition from drug target studies into public databases, such as ChEMBL (Mendez *et al.* 2019), PubChem (Kim *et al.* 2023), and drug target commons (DTC) (Tang *et al.* 2018), still requires manual curation which is labor-intensive and time-consuming. To keep pace with the rapid growth of the literature, automated methods for extracting DTIs are essential for reducing the time and cost associated with drug discovery and development. However, predicting the DTI types from scientific literature remains a demanding task. Our study demonstrates that integrating multiple models within the transformer encoder is essential for optimal performance in mining DTIs from biomedical literature. This approach combines diverse data sources, such as gene descriptions from the Entrez Gene database and chemical descriptions from the CTD, enhancing comprehensiveness and accuracy. The ensemble method, using various pre-trained transformer models like BERT, RoBERTa, BioBERT, and SciBERT, captures different aspects of the data, leading to improved predictive accuracy. Empirical evidence shows that this ensemble approach achieves higher performance metrics, such as an *F*1 score of 80.6 on the hidden DrugProt test set, compared to individual models. Additionally, this integration helps handle complex relationships in biomedical texts and balances trade-offs in precision and recall, making the DTI extraction process more robust and reliable. The proposed methodology significantly improves upon the previous state-of-the-art approaches, showcasing the utility of our approach in biomedical text mining and in enhancing drug discovery efforts. Furthermore, the comparison between the usage of the NCBI Entrez Gene summary and the UniProt protein function description indicates that the gene summary offers more comprehensive information about the DTIs, yielding better model performance.

Several opportunities remain for further enhancements. For instance, our experiments have shown that the inclusion of protein sequence-based features did not yield significant improvement in performance. However, future studies could replace sequence-based features with transcriptomic features as adopted in Xie *et al.* (2018). Furthermore, we have relied on the DrugProt data for validating our model predictions, as DrugProt has been considered the most comprehensive reference dataset for drug–target relationships. In the future, we are planning to manually mine drug–target entities from the 0.3M articles identified in our previous work (Aldahdooh *et al.* 2022). In addition to that, while our method achieved the best performance in the BioCreative DrugProt task, we believe there is still room for improvement in the model architecture. For example, we intend to investigate the potential of leveraging other transformer-based models like BioGPT (Luo *et al.* 2022b).

Lastly, the developed approach and the conclusions drawn are specific to the extraction of DTIs. It would be interesting to investigate the generalizability of this method for other types of relationship extraction applications in drug discovery such as protein–protein interaction and drug–drug interactions. The results from this study thus represent a significant stride in the field of biomedical text mining and drug discovery, and we believe that the continuous advancements in NLP hold immense promise for further progress.

## Supplementary Material

vbae106_Supplementary_Data

## Data Availability

The datasets used in this study are accessible at https://dtis.drugtargetcommons.org/.
